# Geriatric assessment in elderly hemodialysis patients

**DOI:** 10.1590/2175-8239-JBN-2019-0098

**Published:** 2019-08-01

**Authors:** Ertugrul Erken

**Affiliations:** 1 Kahramanmaras Sutcu Imam Universitesi Nephrology Avşar Mah OnikişubatKahramanmaras Turkey Kahramanmaras Sutcu Imam Universitesi - Nephrology Avşar Mah. Batı Çevreyolu Blv. Onikişubat Kahramanmaras 46100 Turkey.

The increasing life expectancy of the world population elicited the increasing numbers of elderly patients starting hemodialysis (HD). Therefore, nephrologists should have detailed knowledge about some issues concerning this elderly population, such as cognitive impairment, frailty, dementia, depression, fall injuries, malnutrition, and polypharmacy, all of which are among the so-called geriatric syndromes.^1^ Although very prevalent among elderly HD patients, these geriatric impairments are usually missed or unheeded. The only way to reveal these geriatric problems is through detailed geriatric assessment.

Cognitive dysfunction and frailty are two of the most important issues in this population since they are both associated with increased comorbidity burden and mortality.^2^ This editorial outlines the importance of geriatric assessment in elderly HD patients for geriatric impairments, particularly cognitive dysfunction and frailty, referring to a recent paper on the topic.^3^

In the study by Viana et al., chronic kidney disease (CKD) patients who started maintenance HD in elderly life were assessed for cognitive functions, mood, and quality of life. The investigators grouped the HD patients as elderly (65-80 years of age) and very elderly (> 80 years of age). Cognitive impairment was more frequent among very elderly patients determined by Mini Mental State Exam (MMSE) and verbal fluency test (VFT). The clock drawing test (CDT) for executive cognitive functions was not different among the groups. They also applied the Geriatric Depression Scale (GDS-15) and the Short Form Health Survey (SF-36) for evaluating the mood and general quality of life, and the very elderly group had worse scores only for functional capacity.^3^ Overall, their results indicated a higher prevalence of impaired cognition and decreased quality of life in very elderly patients with advanced CKD.

Viana et al. demonstrated cognitive impairment in 31.8% of the elderly HD patients group using MMSE. Together with VFT and CDT, the prevalence of having any cognitive deficit reached 71.6% in the elderly group and 93.6% in the very elderly group.^3^ Indeed, using MMSE only may lead to underestimation of cognitive impairment in HD patients who predominantly exhibit cognitive deficits in executive, attention, and memory cognitive domains. Therefore, we should combine MMSE with other cognitive tests like Viana et al. did, or choose a single practical cognitive test that could be more suitable for patients with advanced CKD such as the Montreal Cognitive Assessment (MoCA) (4,5).^4^^,^^5^ After all, testing elderly HD patients for cognitive functions necessitates the selection of a tool that includes evaluation of executive functions. Cognitive testing should be performed before a routine HD session and a suitable cut-off point should be determined by taking into consideration the age and educational status of the subject.^4^ Detection of depressive mood with a practical test like GDS-15 might be a way to eliminate false positivity during cognitive testing in these patients.

Elderly HD patients are a special population that frequently suffer from complications related to comorbid conditions, mostly vascular disease. Geriatric assessment of this population must begin with evaluations for cognitive dysfunction, frailty, and comorbidity burden. Frailty is defined as a decline in physical function and susceptibility to disease-related complications in geriatric populations, and it is more prevalent among patients on HD. In HD patients, frailty is associated with increased hospitalization, vascular disease, and mortality. In addition, frailty is a risk factor for cognitive impairment and fall injuries in elderly HD patients.^2^^,^^6^ Clinical frailty index (CFI) is a practical tool to identify pre-frail and frail patients who have decreased physical activity and physiological reserve in relation to their comorbidities. CFI is also a numerical scale that is useful in grading the severity of frailty.^6^ Comorbidity burden can be assessed with tools like the Cumulative Illness Rating Scale (CIRS) or the Charlson Comorbidity Index (CCI).^4^^,^^7^ It would be wise to reevaluate any HD patient for preventive measures and multidisciplinary treatment strategies when frailty or severe comorbidity burden is detected.

Quality of life questionnaires provide valuable information about the general health status and daily living complaints, but clinicians usually lack enough time to include these tools into everyday clinical practice. There are feasible tools for evaluating quality of life and activities of daily living such as the SF-36 and the Lawton Instrumental Activities of Daily Living (IADL) scale.^3^^,^^7^

There is a growing population of elderly patients with advanced CKD. Besides, CKD itself is considered a state of accelerated aging associated with atherosclerosis, inflammation, cognitive deficits, physical deficits, metabolic abnormalities, and Klotho deficiency. The study by Viana et al. indicates the importance of geriatric impairments in the HD population once again. If we do not pay attention to this unheeded issue, who will? These geriatric syndromes, especially cognitive dysfunction and frail, are impairments that make HD patients dependent on others for activities of daily living.

An accurate geriatric assessment of elderly HD patients necessitates the selection and/or customization of some testing tools. HD patients are in a state of sarcopenia, chronic inflammation, occult cerebrovascular disease, and anemia, which makes them different from general population. Geriatric assessment of elderly HD patients must emphasize evaluations for cognitive impairment, frailty, and comorbidity burden.
